# Forecasted and Observed Drug Overdose Deaths in the US During the COVID-19 Pandemic in 2020

**DOI:** 10.1001/jamanetworkopen.2022.3418

**Published:** 2022-03-21

**Authors:** Abigail R. Cartus, Yu Li, Alexandria Macmadu, William C. Goedel, Bennett Allen, Magdalena Cerdá, Brandon D. L. Marshall

**Affiliations:** 1 Department of Epidemiology, Brown University School of Public Health, Providence, Rhode Island; 2 Department of Population Health, New York University Grossman School of Medicine, New York; 3 Center for Opioid Epidemiology and Policy, New York University Grossman School of Medicine, New York

## Abstract

This cross-sectional study uses data from the National Vital Statistics System to compare forecasted numbers of drug overdose deaths in the US in the latter 43 weeks of 2020 with the observed number of overdose deaths in that period.

## Introduction

The COVID-19 pandemic was associated with the highest annual number of fatal drug overdoses on record in the US in 2020.^1,2^ However, before the pandemic, there was an increasing trend in drug overdose deaths through 2019.^3^ The objective of this study was to estimate the proportion of drug overdose deaths in the US during the latter 43 weeks of 2020 in excess of time series forecasts based on national historical trends.

## Methods

This cross-sectional study used weekly, model-based, provisional overdose death counts using publicly available data from the US Centers for Disease Control and Prevention’s National Vital Statistics System.^4^ Therefore, the study was exempt from institutional review board approval and informed consent by the Brown University institutional review board. The study followed the Strengthening the Reporting of Observational Studies in Epidemiology (STROBE) reporting guideline.

We used estimates of US overdose deaths from February 9, 2016, through March 7, 2020 (corresponding to the World Health Organization declaration of a pandemic on March 11, 2020) to generate forecasts of overdose deaths in the US for the remaining 43 weeks of 2020. We used the forecast package in R, version 4.0.1 (R Project for Statistical Computing)^5^ to fit autoregressive integrated moving average models to the data, manually determining the 3 parameters *p*, *d*, and *q* (where *p* is the autoregressive parameter degree, *d* is the degree of differencing, and *q* is the moving average parameter). These models were then used to generate forecasts for the latter 43 weeks of 2020 (March 8 to December 31). We calculated the total number of forecasted overdose deaths and the proportion of observed deaths in excess of the forecasted deaths during this period. Details of the model building process are provided in the eAppendix in the Supplement.

## Results

Of the 363 086 overdose deaths in the US from 2016 to 2020, a total of 91 799 (25%) occurred in 2020. Our model-based estimates of fatal overdoses showed a steadily increasing trend through 2019 (from 4694 overdose deaths in February 2016 to 5592 in December 2019), a substantial increase in the spring of 2020 (9883 overdose deaths in May), and a subsequent decrease in 2020 (6467 overdose deaths in September) to the level observed at the end of 2019 (Figure).

**Figure.**
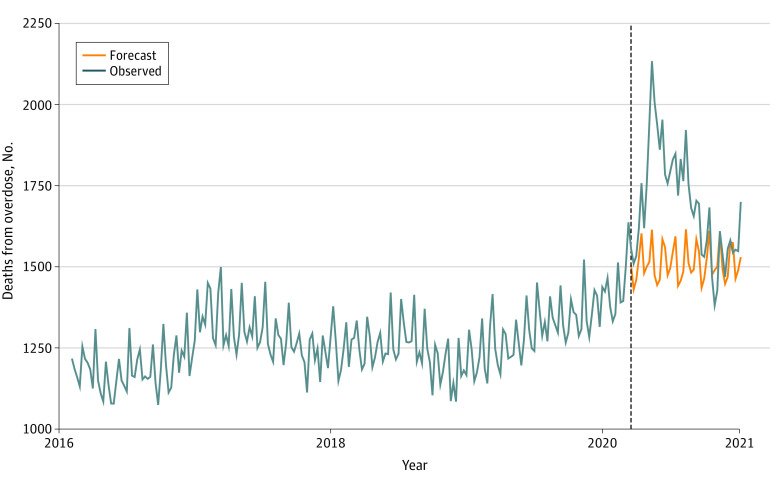
Observed and Forecasted Weekly Model-Based Provisional Estimates of Drug Overdose Deaths in the US From 2016 to 2020 Data from Connecticut and North Carolina were excluded from the analysis. The vertical dashed line indicates the beginning of the forecast.

The forecasted deaths did not substantially increase or decrease from the year-end–2019 level of fatal overdoses (Figure). There were 72 643 overdose deaths observed during the latter 43 weeks of 2020 compared with 65 042 deaths forecasted by our model (difference, 7601 [12%]) (Table). Using the upper and lower prediction limits of the prediction interval of the forecast, plausible forecast scenarios ranged from 56 778 (1%) fewer to 73 306 (28%) more deaths forecasted than observed.

**Table.** Proportion of Observed Overdose Deaths in Excess of the Number Forecasted With ARIMA Models in the US From March 8, 2020, to December 31, 2020^a^

Model^b^	Model type	Total deaths observed, No.	Total deaths forecasted, No. (lower and upper prediction limits)^c^	Proportion of deaths observed to deaths forecasted (lower and upper prediction limits)^c^
ARIMA (7,1,10)	Initial	72 643	65 386 (57 269-73 503)	1.11 (1.28-0.98)
ARIMA (9,1,6)	Final	72 643	65 042 (56 778-73 306)	1.12 (1.28-0.99)

Abbreviation: ARIMA, autoregressive integrated moving average.

^a^ Data from Connecticut and North Carolina were excluded from the analysis.

^b^ The 2 models showed similar performance and are thus presented side by side.

^c^ The prediction limits are for the forecasts generated by each model.

## Discussion

In this cross-sectional modeling study, we found that fatal overdose deaths in the US in the latter 43 weeks of 2020 exceeded our model’s forecasts by 12%, or 7601 deaths, with plausible forecast scenarios spanning no deaths in excess of the forecasted number to observed deaths 28% in excess of the forecast.

 A limitation of this study is that the data did not include estimates from North Carolina or Connecticut (states with substantial lags in death reporting) and therefore are not nationally representative.^4^ Second, the differences between the Centers for Disease Control and Prevention’s provisional and final data are small^2^; however, because we used model-based provisional estimates and not final counts, any error present in the estimates would be propagated through the forecast. Third, our forecasts represent just 1 possible counterfactual scenario for overdose mortality in the absence of the pandemic; because our forecasted mortality was flat, our estimates of excess mortality may be exaggerated. We conducted several sensitivity analyses, and the forecasted mortality remained flat unless problematic modeling assumptions were made. Furthermore, our results captured mortality in excess of our forecast for the remainder of 2020, not in excess of overdose mortality in 2019.

Our forecasts represent a counterfactual scenario in which overdose deaths stabilized at a high level from March to December 2020. Although overdose mortality steadily increased throughout 2019, our model’s forecast did not yield a continued secular increase through 2020. Although the trend in overdose mortality if the pandemic had not occurred cannot be known, based on historical time trends, this study’s findings suggest that the pandemic may have been associated with increased overdose mortality in the US in 2020. Although the prediction limits of our forecast indicated a scenario of no excess overdose mortality, our finding of mortality exceeding the forecast is consistent with emerging national data. This study’s results are consistent with the emerging consensus that the COVID-19 pandemic was associated with increased overdose mortality in 2020.^6^ Our findings further underscore the importance of swiftly controlling transmission of infectious diseases to minimize disruptions to treatment of substance use disorders and access to harm reduction services.

## Notes

Supplement.
eAppendix.
