# Supplementary material for: Forecasted and Observed Drug Overdose Deaths in the US During the COVID-19 Pandemic in 2020
Source: JAMA Netw Open. 2022 Mar 21;5(3):e223418. doi: 10.1001/jamanetworkopen.2022.3418 (PMC8938716; doi:10.1001/jamanetworkopen.2022.3418)
Supplement: Supplement. — eAppendix. [file jamanetwopen-e223418-s001.pdf]

Supplementary Online Content

Cartus AR, Li Y, Macmadu A, et al. Forecasted and observed drug overdose deaths in the US during the COVID-19 pandemic in 2020. *JAMA Netw Open*. 2022;5(3):e223418. doi:10.1001/jamanetworkopen.2022.3418

eAppendix.

This supplementary material has been provided by the authors to give readers additional information about their work.

© 2022 Cartus AR et al. *JAMA Network Open*.

Supplemental information: methods

Data source

The data we used for this analysis were National Center for Health Statistics (NCHS) "nowcasting" model-based provisional estimates. The purpose of these estimates is to improve the timeliness of reporting for several causes of death, including overdose. Final mortality estimates are typically not available for approximately 12 months following the close of a given calendar year. Provisional estimates, an important component of mortality surveillance, improve on this timeliness somewhat – provisional estimates are typically available 6-9 months after the date of death. NCHS has employed these "nowcasting" methods to improve further upon the timeliness of provisional estimates and to generate more up-to-date estimates of overdose mortality through 2020.

In order to generate the "nowcasting" estimates, NCHS investigators modeled completeness of weekly provisional data from 2018-2019, then used posterior predicted values of completeness to generate weights, which were then applied to new sets of models to generate posterior predicted weekly counts of deaths for each jurisdiction, including nationally for the United States. The national estimates cover 48 states, with Connecticut and North Carolina excluded because of long reporting lags for overdose mortality. The NCHS investigators modeled a range of scenarios reflecting different assumptions about possible changes in timeliness of death reporting, which are explored at length in Rossen *et al.* (2021). The investigators also validated the "nowcasting" estimates against provisional estimates and found good agreement. Since more provisional estimates are now available, it is possible to check the predicted against the provisional estimates. At the time of writing, agreement through January of 2021 still appears to be good.

Model building

We first inspected the time series visually and conducted Friedman tests to assess seasonality and augmented Dickey-Fuller tests to determine the appropriate order of "differencing" (*d* parameter) to achieve stationarity. We observed no evidence of seasonality in the time series from 2016-2020. The time series had a non-stationary pattern evident upon visual inspection and with formal statistical testing (augmented Dickey-Fuller test p-value = 0.89) which one order of differencing was sufficient to resolve.

We inspected autocorrelation and partial autocorrelation function (ACF/PACF) plots to determine the value of the autoregressive (*p*) parameter, then evaluated a range of moving average (*q*) parameters (0-18) with the fixed values of the other parameters. We chose the model with the smallest AIC as our final model. The number of lags indicated by the ACF plot of the time series was borderline, so we first chose the autoregressive parameter conservatively (7 lags). The ARIMA(7,1,*q*) model (*q* ranging from 0-18) with the lowest AIC was ARIMA(7,1,10) (AIC = 2259). A Ljung-Box test of the residuals from this model indicated some remaining autocorrelation among the residuals (p-value = 0.03).

Consequently, we engaged in another round of model building. We increased the autoregressive parameter to 8, 9, and 10, and with one order of differencing evaluated models with these higher

© 2022 Cartus AR et al. *JAMA Network Open*.

autoregressive parameters with moving average parameters ranging from 0-18. We selected the model with the lowest AIC from this process (ARIMA(9,1,6), AIC = 2256). A Ljung-Box test of the residuals from this model indicated less potential for autocorrelation among the residuals (p-value = 0.08). As a result, this model was chosen as our final model. Results generated with the other model explored are presented in Table 1 and are highly consistent with results from the final model.

Forecasting

The forecasting software program we used (*arima()*) generates a forecast for each week with corresponding upper and lower confidence bands. We used these upper and lower confidence band to construct pseudo-confidence intervals around the forecasts for each week. We were also able to use the upper and lower confidence estimates to produce upper and lower estimates of the total number of forecasted overdose deaths in 2020. These estimates represent the range of plausible forecasts based on the data, ranging from more conservative to less conservative estimates. Whether the forecast or the upper or lower confidence estimates are used affects the degree of overdose mortality in excess of the forecast, with the upper estimate producing a more conservative estimate (lower overdose mortality in excess of the forecast) and the lower estimate a less conservative estimate (higher overdose mortality in excess of the forecast).

References

Rossen LM, Hedegaard H, Warner M, Ahmad FB, Sutton PD. Early provisional estimates of drug overdose, suicide, and transportation-related deaths: Nowcasting methods to account for reporting lags. Vital Statistics Rapid Release; no 11. Hyattsville, MD: National Center for Health Statistics. February 2021. DOI: http://dx.doi.org/10.15620/cdc:101132.

© 2022 Cartus AR et al. *JAMA Network Open*.
